# Bonnie and Clyde: Vitamin C and iron are partners in crime in iron deficiency anaemia and its potential role in the elderly

**DOI:** 10.18632/aging.100966

**Published:** 2016-05-16

**Authors:** Darius J. R. Lane, Patric J. Jansson, Des R. Richardson

**Affiliations:** Department of Pathology and Bosch Institute, Molecular Pharmacology and Pathology Program, Blackburn Building, University of Sydney, Sydney, New South Wales, 2006, Australia

**Keywords:** iron, ascorbic acid, aging, erythropoiesis, anaemia

Iron is an obligate nutrient for life, due to its key roles in myriad cellular processes. This vital transition metal is also an essential component of haemoglobin in red blood cells [3, 4], of which 200 billion new red cells are produced every day [5, 6].

It is well known that vitamin C (ascorbate) is crucial for collagen formation and the prevention of scurvy [2]. However, an underappreciated fact is that ascorbate, in addition to its well established role in dietary iron absorption, is also vital for maximal uptake of iron from the serum-iron transport protein, transferrin [1, 2] (Figure 1).

**Figure 1:**
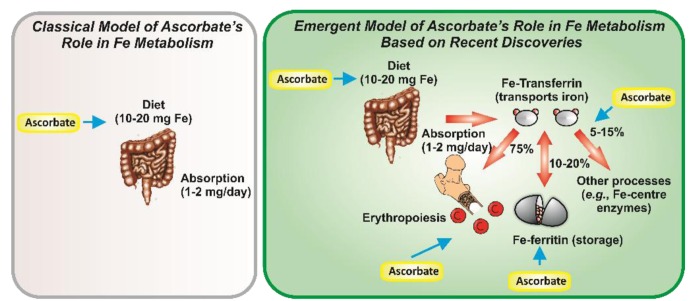
Ascorbate was thought to play a limited, simple role in dietary Fe uptake (left). Recently, we have proposed an integrated role for ascorbate in enhancing tissue Fe uptake & metabolism (right) [1, 2].

Indeed, in light of these new findings, the role of combination therapy employing both iron and ascorbate requires critical reappraisal for the treating iron-deficiency anaemia. The following epidemiological observations are pertinent to this discussion: (1) while ascorbate deficiency is thought to be rare in Western countries, it is surprising that 7.1% of US adults and up to 40% of elderly UK individuals (≥65 years) were ascorbate-deficient [7-9]; (2) low ascorbate is common in critical care patients[10], including Australian dialysis patients [11]; (3) ascorbate deficiency remains a critical problem in the third world, and also in indigenous populations, *e.g*., Australian Aborigines [12]; and (4) by age 80 years, up to 10% of Australians are anaemic, and this contributes to significantly increased risk of disability, morbidity and death [13]. Thus, optimising combinations of iron and ascorbate could be vital.

Anaemia in the elderly is of high clinical relevance, as several studies have linked it to an increase in overall morbidity, Alzheimer's disease [14], decreased “quality of life” scores, and increased rates of hospital admissions and mortality [15]. In considering the causes of anaemia in the elderly, it is worth noting that chronic inflammation is common in elderly individuals, which increases circulating hepcidin levels, leading to a suppression of circulating iron that is available for erythropoiesis [16]. Other significant contributory factors to anaemia in the elderly are a poor diet, which may be deficient in iron, folate and/or ascorbate, and may potentiate or promote iron deficiency and iron deficiency anaemia (IDA) [16].

As Lopez and colleagues [17] recently reported, anaemia affects approximately one third of the global population, namely 2.1 billion people, with IDA accounting for at least half of these cases [17]. In fact, IDA represents a health problem of global proportion, with the burden of disease falling on young children (0-5 years of age), women of childbearing age, pregnant women and the elderly [17]. Worryingly, iron deficiency has also emerged as a significant co-morbidity and therapeutic target of chronic heart failure, chronic renal insufficiency, anaemia and type 2 diabetes [18].

Importantly, Lopez and colleagues [17] discuss strategies for acute and long-term management of IDA, with the ultimate goal being to “…supply enough iron to normalize haemoglobin concentrations and replenish iron stores, and thereby improve quality of life” [17]. Although the role of ascorbate in increasing dietary iron absorption is mentioned by these authors in reference to its ability to enhance iron bioavailability [17], unfortunately, in such discussions the important role of ascorbate in facilitating transferrin-dependent iron uptake is frequently overlooked [1, 2, 19]. This pathway of cellular iron uptake is essential as it provides the sole source of iron for erythropoiesis [3]. The frequent under-recognition of the emerging interplay between ascorbate and iron utilisation post-absorption is concerning, given that severe ascorbate deficiency leads to pronounced anaemia in mice [20], and is associated with anaemia in humans [2].

In support of the aetiological connection between ascorbate deficiency and anaemia, it was recently demonstrated that physiological ascorbate concentrations cause a marked increase in iron uptake from transferrin in a wide variety of cell-types in culture [1]. It is well described that during endocytosis of the transferrin-iron/transferrin receptor 1 complex by erythroid cells [3, 4], ferric iron must be reduced to ferrous iron prior to traversing the endosomal membrane into the cytosol [19].

In fact, a source of reducing equivalents is required for iron release from transferrin-containing endosomes [3, 4], with ascorbate being quantitatively more significant than NADH [19]. This concurs with the finding that, although the only identified ferrireductase of the transferrin cycle is the NAD(P)H-dependent enzyme, STEAP3, *Steap3^−/−^* mice have 60% of the haemoglobin levels of wild-type mice [2]. Thus, transferrin iron must be able to be reduced by other means, which appear to include intracellular ascorbate [1, 2, 19].

It is also worth discussing that Lopez and colleagues [17] state that, while ascorbate can improve the bioavailability of dietary iron, it also increases the frequency of adverse gastrointestinal effects of oral iron supplements, such as “…epigastric discomfort, nausea, diarrhoea, and constipation, because it increases the amount of ferrous iron downstream”. It is critical to point out that such effects typically result from the promotion of iron-driven oxidative stress (*i.e*., Haber-Weiss-like reactions) in the gut lumen. Such oxidative reactions are known to prevail at low ascorbate-to-iron ratios [21], but can be circumvented by increasing this ratio [21].

Optimising the ascorbate-to-iron ratio in iron supplements should lead to quenching of iron-driven radical production [21]. This simple and low-cost alteration to iron supplements containing ascorbic acid could significantly diminish adverse gastrointestinal effects. Additionally, an increase in the ascorbate-to-iron ratio would be expected to promote increased iron solubility, due to combined reductive and chelation effects, which would enhance iron absorption and downstream utilisation.

Such intricacies of the ascorbate and iron interaction are vital to consider, especially given the recent finding that fortification of infant formula can lead to serious effects on the infant gut microbiome [22]. As such, appropriately increased ratios of ascorbate to iron in iron supplements may lower the total level of iron required to achieve the same desired clinical endpoint.

In conclusion, the critical, stimulatory role of ascorbate in iron uptake from transferrin [1, 2], the sole source of iron for erythropoiesis, is important to consider in relation to better understanding and effectively treating the rampant global problem of IDA, the burden of which falls on heavily at-risk groups such as the very young, pregnant women and the elderly [17].

## References

[1] Lane DJR (2013). Biochim Biophys Acta.

[2] Lane DJR, Richardson DR (2014). Free Radic Biol Med.

[3] Dunn LL (2007). Trends Cell Biol.

[4] Richardson DR (2010). Proc Natl Acad Sci USA.

[5] Kalinowski DS (2016). Biochim Biophys Acta.

[6] Lane DJR (2015). Biochim Biophys Acta.

[7] Elia M, Stratton RJ (2005). Nutrition.

[8] Mandal SK, Ray AK (1987). J Int Med Res.

[9] Schleicher RL (2009). Am J Clin Nutr.

[10] Berger MM, Oudemans-van Straaten HM (2015). Curr Opin Clin Nutr Metab Care.

[11] Singer R (2008). Nephrology.

[12] Ben-Zvi GT, Tidman MJ (2012). Practitioner.

[13] Chalmers KA (2012). Age Ageing.

[14] Faux NG (2014). Mol Psychiatry.

[15] Aspuru K (2011). Int J Gen Med.

[16] Fairweather-Tait SJ (2014). Mech Ageing Dev.

[17] Lopez A (2015). Lancet.

[18] Cohen-Solal A (2014). Heart.

[19] Nunez M-T (1990). J Biol Chem.

[20] Maeda N (2000). Proc Natl Acad Sci USA.

[21] Buettner GR, Jurkiewicz BA (1996). Radiat Res.

[22] Jaeggi T (2015). Gut.

